# “It is really just brilliant to get credits for something that is so important to you!” *Skills for Life*: University students’ perceptions of a planned dietary life skills course

**DOI:** 10.1371/journal.pone.0260890

**Published:** 2022-04-07

**Authors:** Ida Ulrikke Valand, Nina C. Øverby, Sofia Strömmer, Mary Barker, Camilla Bjornes, Julie Nordli, Line Pettersen, Tormod Bjørkkjær, Frøydis N. Vik, Charlotte Kiland, Elisabet R. Hillesund

**Affiliations:** 1 Department of Nutrition and Public Health, Faculty of Health and Sport Sciences, University of Agder, Kristiansand, Norway; 2 MRC Lifecourse Epidemiology Unit, University of Sout,lhampton, Southampton General Hospital, Southampton, United Kingdom and NIHR Southampton Biomedical Research Centre, University of Southampton and University Hospital Southampton NHS Foundation Trust, Southampton, United Kingdom; 3 School of Health Sciences, Faculty of Environmental and Life Sciences, University of Southampton, Southampton, United Kingdom; 4 Department of Upbringing and Culture, Bjerkreim Municipality, Bjerkreim, Norway; 5 Department of Political Science and Management, Faculty of Social Sciences, University of Agder, Kristiansand, Norway; International Hellenic University: Diethnes Panepistemio tes Ellados, GREECE

## Abstract

**Objective:**

Universities have a role in educating and empowering students to become healthy and literate citizens of the 21^st^ century society. The aim of this study was to explore university students’ perceptions regarding the relevance and utility of a planned dietary life skills course.

**Design:**

Qualitative design including focus group discussions.

**Setting:**

A Norwegian university with participating undergraduate students from seven different disciplines.

**Method:**

Data collection included 13 semi-structured focus group discussions involving 57 university students (35 women and 22 men aged 18–38 years). The focus group discussions were recorded and transcribed verbatim. To ensure in-depth knowledge of the research participants’ thoughts and reflections, thematic analysis strategy was undertaken by a team of researchers.

**Results:**

When presented to the idea of a dietary life skills course as a university course, the students were mostly positive regarding its relevance and utility, however both motivators and barriers for attending were put forward. Some mentioned potential academic course benefits, such as enhanced CV, and a few mentioned potential societal benefits such as a healthy population and sustainable food consumption. Several motivators for attending the course were launched, such as increased knowledge and cooking skills, having dinner and expanded network. The students wanted to learn about food, nutrients and health, and how to cook simple, affordable, healthy and sustainable meals. Potential barriers for attending were mostly related to practicalities, such as potential lack of alignment with ordinary study programme or too demanding lectures.

**Conclusion:**

Most students acknowledged the value of a dietary life skills course and thought that such a course could benefit their personal life. This encourages the offering of such courses at university level, tailored to consider both motivators and barriers for attending.

## Introduction

Universities have a role in their students’ lives beyond just providing higher-level education to secure a degree within a specific discipline. The education they provide is more holistic; promoting general competencies like literacy, citizenship and character development [1, 2]. Young adults like students should be prepared for a changing world and future challenges, both concerning working and personal life and their participation in society. The concept of life skills in higher education has become more recognised internationally, with many experts calling for better integration into existing curriculums at all education levels [3, 4]. Building on this momentum, the interdisciplinary topic of ‘health and life skills’ has recently been implemented in all curriculums in compulsory education in Norway. Life skills are transferable skills that enable individuals to cope with, and make the most out of life, progress and feel capable at school, work and in society [5]. These skills comprise the interaction of attitudes, values, behaviours, knowledge and abilities, and harmony in this interaction is supportive of a sense of empowerment and an enhanced quality of life [6]. For example, life skills promote literacy, critical thinking, and communication skills [7], and are also directly linked to our health, and shape our risk of developing diseases later in life [8, 9]. With poor diet as a leading cause of both global and national burden of disease [10, 11], life skills that can achieve and maintain a healthy diet throughout life are key in determining health at a societal level. In Norway, almost 90% of the national disease burden relates to non-communicable diseases (NCDs) such as obesity, type 2 diabetes, cardiovascular disease, cancer, and mental disorders [12]. As NCDs are complex and difficult to treat, better strategies for primary prevention are warranted [13, 14].

Life skills can be learnt throughout life, but interventions targeting specific skills are likely to be more effective at specific life stages [5]. The transition from living with one’s parents to university life represents a considerable life change that has been associated with deteriorated health and health behaviours [15, 16] such as weight gain [15, 17–19] and adverse dietary changes including increased alcohol and sugar consumption, and reduced intake of fruit and vegetables [15, 20]. In Norway, a recent study among university students revealed that they ate substantially less than recommended amounts of fruits and vegetables and fish [21]. Supporting the development of life skills during these transitions can enhance the health and wellbeing of young adults in the present and improve their health status in later life.

As potential prospective parents, young people in their preconception years also hold the key to health in the next generation. Recent compelling evidence shows that the diet of prospective parents is not only important for their own lifelong health, but also fundamental to the lifelong physical and mental health of their children [22, 23].

One way of promoting the attitudes, values, behaviours, knowledge and abilities needed to achieve and maintain a healthy diet could be through offering a dietary life skills course at higher education institutions, such as universities. Through such a course, students may develop the knowledge, tools and skills that are necessary for better dealing with their diet-related health and wellbeing in the transition from living with their parents into building a life on their own or with a partner. By strengthening diet-related life skills, the students may improve their immediate and long-term health and wellbeing, and consequently their prospective children’s health and wellbeing as well. In this way, a dietary life skills course may be viewed as a public health initiative with potential transgenerational impact [24, 25]. For such an initiative to be effective and have lasting health impact, however, it needs to be developed in close co-creation with the target group [26]. Therefore, we aimed to gain in-depth insight into university students’ perceptions of the relevance and utility of a proposed university course targeting dietary knowledge and skills.

## Methods

### Design

In this qualitative study, 13 semi-structured focus group discussions (FGDs) with a total of 57 university students (35 women and 22 men) were conducted to explore their views on a proposed dietary life skills course and potential barriers and facilitators to engagement, as a means of co-creation. The students were informed that information from the FGDs could be used to develop an elective, credit-providing course, available for all students at their university, focusing on nutrition, health and food preparation skills, aiming to enhance students’ knowledge, attitudes and skills regarding food and health. The Skills for Life course is planned to be piloted in 2023. The students were also informed that their opinions were recorded for research purposes with the aim to be published in an academic journal. The FGDs were conducted in September and October 2019 at a Norwegian university (University of Agder, Department of Nutrition and Public Health) with approximately 13 000 students and seven disciplines (i.e., Business and Law, Engineering and Science, Fine Arts, Health and Sport Sciences, Humanities and Education, Social Sciences, Teacher Education). The focus groups comprised undergraduate students from all faculties/disciplines at the university. Data was analysed using thematic analysis and the study adopted a relativist ontological and subjective epistemic position, rooted in the belief that reality is always constructed relative to a particular frame of reference and influenced by personal experience and insight [27, 28]. The study is reported following the Consolidated Criteria for Reporting Qualitative Research [29]. The project was notified to NSD–the Norwegian Centre for Research Data and approved by the University of Agder Faculty of Health and Sport Science’s Ethical Committee. All procedures contributing to this study comply with the ethical standards of the Helsinki Declaration of 1975, as revised in 2013.

### Procedures

**Fig 1:**
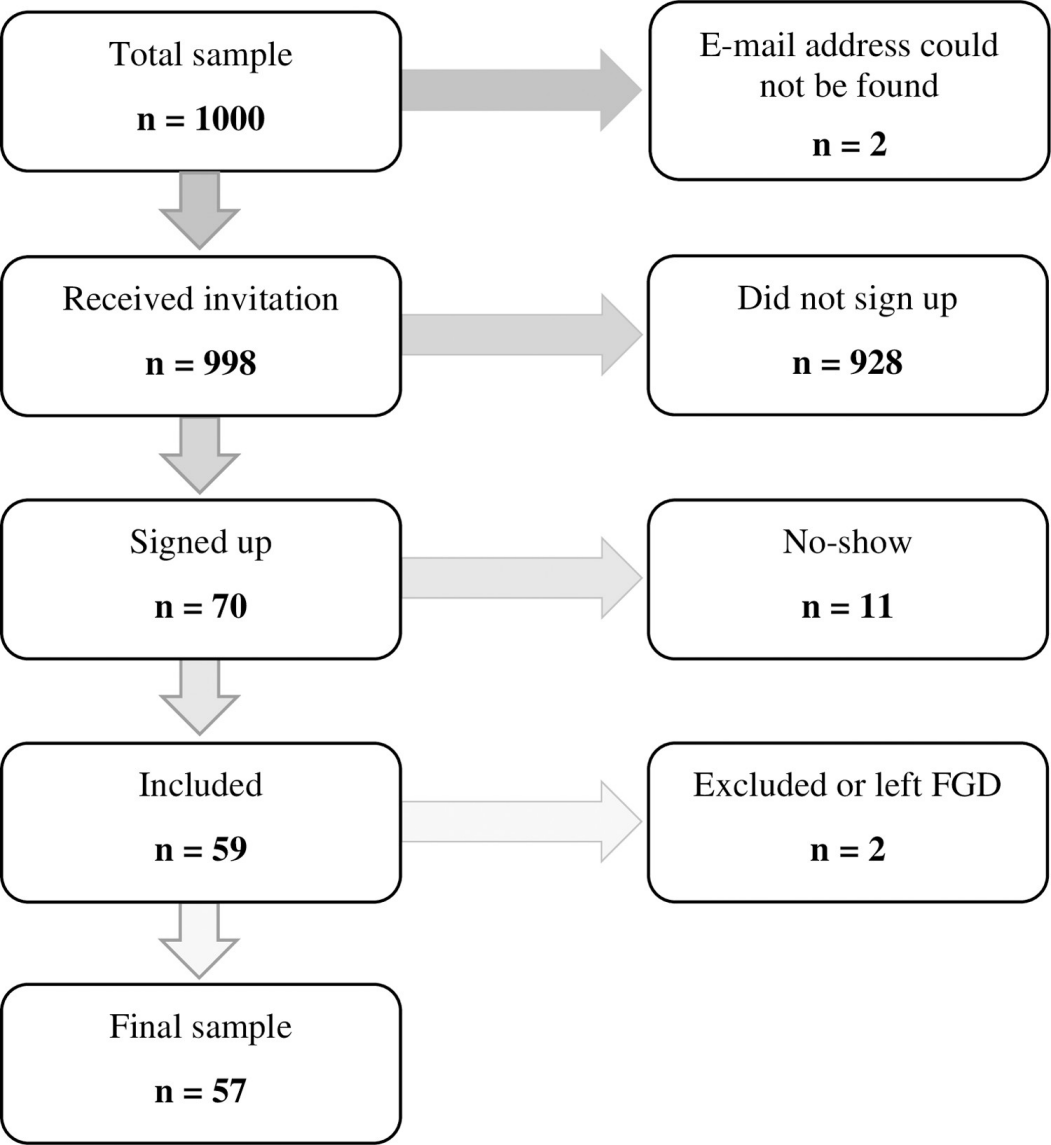
Recruitment process flowchart. FGD, focus group discussion.

The FGDs were led by three postgraduate students in Public Health (CB, JN and LP) who used this data for their MSc dissertations, each focusing on one of three topics: students’ reflections on preconception diet in relation to future health, determinants of food choices and diet quality, or sustainability issues related to diet. As such, the target sample was 60 participants to ensure sufficient data to cover these three topics in addition to the cross-cutting topic covered in the present paper. Approval to recruit through the students’ institutional e-mails was obtained from the university management, and a list of 1000 students at the university (500 men and 500 women) was provided by administrative staff. The inclusion criteria were: undergraduate student aged 18–40 years. As the information sheet was written in Norwegian, non-Norwegian-speaking students were excluded. The invitation e-mail was sent to the 1 000 students in September 2019, and contained information about the project and participation, and a link to the project website. The e-mail also stated that participants would receive a voucher of NOK 200 for the university cafeteria. The website provided complementary information and a link to online registration through SurveyXact. Students signed up by choosing from a list of available FGD times and provided written informed consent using the online registration form. A reminder was sent once to those who had not enrolled the week after sending the invitation e-mail. The flow of participants is presented in Fig 1.

Focus groups consisted of 2–6 students and 10 out of 13 groups included both men and women. Each FGD were conducted in Norwegian, they took place in a group study room on campus and lasted between 50 and 90 minutes. The postgraduate students’ supervisors (ERH and TB) advised on how to conduct FGDs, and CB attended a qualitative training course on behalf of the interview team. Each FGD was facilitated by one researcher (postgraduate student) and observed by the other two. Each researcher thus facilitated four or five FGDs and acted as an observer in the others which involved listening and taking notes. The first part of the FGDs focused on the research topic of the facilitating researcher: preconception diet in relation to future health, food determinants, or sustainable diet. The last part of each FGD focused on the perceived relevance and possible content of a planned life skills course targeting diet. The data for the present analysis was retrieved from this last section of the FDGs, comprising 10–15 minutes of each FGD.

A semi-structured interview guide was used to facilitate the 13 FGDs in a consistent way and to ensure coverage of the relevant questions. In preparation for the FGDs each researcher facilitated a practice FGD prior to formal data collection to develop their skills and ensure the suitability of the interview guide and make any necessary adjustments. In the last part of the FGD, the facilitator introduced the idea of an elective diet and nutrition course (hereafter *Skills for Life*), as described above. The FGD comprised open-ended questions such as: what do you think of such a course, in which way would you prefer the course to be organised, what themes do you wish to know more about and what would it take for you to attend the course. Follow-up questions were asked when appropriate.

The FGDs were audio recorded using two Institutionally approved dictaphones without internet connection, in line with General Data Protection Regulation [30]. Both dictaphones were used in all FGDs in case one went out of battery. Recordings were transcribed verbatim and cleaned by CB, JN and LP. Transcription and cleaning were performed by different researchers to ensure accuracy before the recordings were deleted. To secure anonymity, participants were given ID numbers (e.g. 1A for interview 1, student A) in the transcripts. The ID numbers were also used to accompany verbatim quotes in the results section of this article.

The transcripts related to *Skills for Life* were analysed using systematic text condensation strategy [31]. This method of thematically analysing qualitative data consists of a systematic step by step guide of the analysis process: 1) total impression–from chaos to themes, 2) identifying and sorting meaning units–from themes to codes, 3) condensation–from code to meaning, and 4) synthesising–from condensation to descriptions and concepts [31]. After reading the transcripts, IUV and ERH focused on the first four interviews and independently wrote down notes regarding the questions in the interview guide. Starting with a small part of the data material corresponds to the text condensation strategy and is helpful to get an overview of the data [31]. After working on the notes and rereading the four transcripts independently, IUV and ERH discussed the notes and agreed upon four preliminary themes that were written down: perceived relevance/benefit, learning outcomes/course content, prerequisites for success (student attendance) and potential challenges/barriers to wanting to attend the course. Further analysis was done by IUV, supervised by ERH, CK and NCØ. All 13 transcripts were colour-coded into themes in Microsoft Word. In the next step, meaning units (i.e. transcript fragments that relates to the research question [31]; usually short or long sentences) from each theme were coded into different subgroups within each of the four overarching themes. If a meaning unit fitted into more than one theme or subgroup, it was coded into all appropriate groups. This coding was performed in qualitative data analysis software NVivo 12. Preliminary themes were translated to English and discussed with senior members of the team at a qualitative training course, and the revised themes and subgroups are presented in the results section. Discussions with senior team members focused on the deeper meaning of participant accounts as represented by the themes, and the implications for the development of a diet-related life skills course.

In line with text condensation strategy, all meaning units in each subgroup were put together and condensed into statements, illustrated by verbatim quotes. In the final step, the main content of these texts was translated to English and synthesised to be presented in a descriptive way in the results section. The results were compared to the transcripts to ensure compliance with the participants’ voices.

## Results

Participants were 18–38 years of age with most participants being in their twenties. Twelve of the 13 focus groups contained students from multiple disciplines. Focus group characteristics are presented in Table 1.

**Table 1:** Focus group characteristics.

Focus group	Primary Topic	Participant gender and age	
1	Sustainable diet	2 men	23–26 years
2	Preconception diet	4 women, 1 man	20–25 years
3	Food determinants	2 women	23–27 years
4	Food determinants	3 women, 2 men	19–30 years
5	Sustainable diet	5 women, 1 man	20–24 years
6	Preconception diet	3 women	23–27 years
7	Food determinants	3 women, 2 men	19–31 years
8	Sustainable diet	2 women, 2 men	19–38 years
9	Sustainable diet	5 women, 1 man	18–32 years
10	Preconception diet	1 woman, 3 men	21–30 years
11	Preconception diet	2 women, 2 men	19–23 years
12	Food determinants	2 women, 3 men	20–24 years
13	Sustainable diet	3 women, 3 men	19–23 years

In the thematic analysis of the FGDs we identified the following themes: 1) Perceived benefits of a *Skills for Life* course, 2) perceived motivators and 3) barriers for attending, and 4) what kind of course content or learning outcomes students wanted.

### Perceived benefits of a *Skills for Life* course

Students taking part in this study described three kinds of potential benefits of attending the proposed *Skills for Life* course: 1) personal, 2) academic, and 3) societal. Personal benefits were most predominant and mentioned by students from all disciplines except Humanities and Education, especially Fine Arts and Health and Sport Sciences. The benefits are described below with illustrative quotes.

#### Personal benefits

Overall, the proposed *Skills for Life* course was perceived as an interesting and useful initiative that could be a fun, inspirational or motivational variation to other university courses. Personal benefits of attending, such as learning about nutrition and cooking, eating more sustainably, saving money, and improving health were described. *[…] it is really just brilliant to get credits for something that is so important to you […]* (3A, female, 23, Health and Sport Sciences). It was said that particularly students who have not lived by themselves for very long would benefit from acquiring good habits that could eventually be transferred to future generations. One student asserted that many students are unaware of how to live an “adult” life, in the sense that they do not know how to eat healthily and live good lives and are ignorant of the long-term consequences of a poor diet. Another student felt that there was no way of learning such skills elsewhere, hence *Skills for Life* could benefit students by providing tools and promoting skills on how to live “adult” lives and make good decisions. Some students claimed that knowledge related to food and cooking was as important for their life as the subject they studied, and something that students might need to improve their quality of life. *I think it would be very rewarding for most people*, *not just because of the credits; it is life knowledge that one acquires* (6C, female, 23, Health and Sport Sciences). It was emphasised that dietary knowledge is important for everyone, regardless of profession; to live good lives and perform at work. The students appreciated the opportunity to learn more than they could read online and thus becoming able to critically assess the information online. Some students valued the opportunity to take such a course intended for personal use rather than having to study nutrition, as they would not go on to take a nutrition degree, but still wanted to acquire more knowledge on the topic. One student reflected on the fact that the *Skills for Life* topic corresponds with people’s values today; that focusing on diet is a little “hip”. It was claimed that they would attend the course if offered, e.g. to learn or because they thought they did not know enough about this topic.

#### Academic benefits

The *Skills for Life* course was perceived as a relevant and useful complement to a number of studies, e.g. nursing, social work and teacher education, and it was said that the course could be implemented in such study programmes. *I think there are many studies that could benefit from it as a compulsory course*. *Well*, *kindergarten teacher*, *for school*, *child welfare* (9C, male, 32, Social Sciences). The possibility of collaborating and interacting with students from different academic fields and study programmes was highlighted as beneficial to their future careers and in their present academic lives. The course would have to be included on their diploma or CV, and several students hoped that this would give them an advantage when applying for jobs, e.g. nurse or social worker. *[…] when applying for a job I would know that ok*, *this [course] would be a plus* (6A, female, 23, Health and Sport Sciences).

#### Societal benefits

The course was highlighted as a public health initiative, and the importance of diet to both health and economic growth was brought forward. *You study to like get a job*, *but also–you have to live the life you live too*, *so to speak*, *and diet has very much to say also to stay in work too*. *Like*, *I am not only thinking about our children*, *so to speak*, *but also as an economics student*. *The economic growth*, *health has a lot to say* (6C, female, 26, School of Business and Law). It was claimed that many people in general lack knowledge regarding diet and health, and date labelling in relation to food waste. Sustainable food choices and learning how to use one’s senses in order to reduce food waste was emphasised as important knowledge that could benefit society.

### Perceived motivators for attending a *Skills for Life* course

Four key motivators for attending the *Skills for Life* course were identified: 1) cooking lessons, 2) fun and rewarding, 3) schedule and ambitions, and 4) tailored and instructive marketing.

#### Cooking lessons

Cooking lessons was highlighted as an important motivator for attending the proposed *Skills for Life* course. *[…] I just think*, *in general*, *the offer of learning how to cook cheap*, *sustainable food would be very attractive to students […]* (13E, female, 19, Health and Sport Sciences). Furthermore, having dinner in course sessions would make it easier to prioritise spending afternoon time at the university. It was said that most people know that vegetables are healthy, but how to prepare the vegetables would possibly be more useful to know, and something that might help the students in their everyday life. It was mentioned that many students lack cooking experience, and that *Skills for Life* participation could enhance their diet and improve their economy. *I think people would choose the subject just for the sake of improving their economy and their way of eating*, *across all study programmes* (13C, male, 21, Social Sciences).

#### Fun and rewarding

The students highlighted that a practical, interactive and well composed course with well-connected theory and practice would be engaging. They thought that advertising for the course should emphasise that you get something out of it, e.g. credits or an edge to the CV, and that it would help students here and now, especially financially, and by improving diet and their own health. It was advised that the course should be promoted as something that enhances achievements in other university courses and thus has the potential to improve grades. If the course were to be offered early in the first semester, students might attend to get to know new people. It was said that the course ought to be challenging and something they needed to strive for, and participants thought that more students might be willing to attend if the course provided 10 credits and thus represented a full course. *I would have considered taking it more strongly if I felt that there was certainty that I could learn something new*, *whether food from other cultures or brand new ways of doing things*. *Because I feel that learning new skills*, *or the theory*, *I can sort of do that on my own*. *So then they had to offer something that I cannot google by myself in a way* (7E, female, 29, Social Sciences).

#### Schedule and ambitions

If the students were to attend the *Skills for Life* course, it would have to fit their schedule. Some said that the probability of attending would increase if the course had been compulsory in their course plan, while others were motivated by the possibility of the course to replace another university course, or to choose it without missing other classes of importance. Some said that they would attend if the classes were scheduled occasionally, e.g. one afternoon every week or fortnight. *So*, *it must be arranged so that you can come when it actually fits into your schedule* (11D, male, 19, Engineering and Science). It was also reflected that by using pass/fail instead of grades, the threshold of attending would be lower, hence increasing the potential for students to learn about healthy lifestyle.

#### Tailored and instructive marketing

The students emphasised the necessity of sufficient and helpful information about the course being provided well before course start, so that they knew of its existence and what it was about. The marketing should include that the course would give a high dividend. *Sell it*! one student (3A, female, 23, Health and Sport Sciences) exclaimed. It was stated that *Skills for Life* should be very low-threshold and embrace all kinds of people, regardless of prerequisites such as prior knowledge and main study programme. *Yes*, *and that it is very like low-threshold*, *I believe is important*. *But also that you get good information about what it actually involves*, *because I think many may think that “no*, *it is not relevant to me*, *it is not something that I am interested in”*. *But if you get the right information about what the course actually involves*, *I guess more people would choose it* (11C, female, 23, School of Business and Law).

### Perceived barriers for attending a *Skills for Life* course

Three kinds of potential barriers for attending were identified in the analysis: 1) lack of relevance, 2) lack of appeal, and 3) too demanding, demotivating, or expensive.

#### Lack of relevance

Some students did not see the relevance of the proposed course to the subject they studied and would rather prioritise more relevant courses. *[…] many would think that “this is not what I study to become*, *so why should I take it when I can take another course*?*” […] “I know this*. *I can figure it out by myself”* (11B, male, 23, Humanities and Education). Furthermore, some questioned whether those who actually need the course, rather than those who already take interest in diet and nutrition, would attend. *But many of those who would choose such a course would probably already be very interested and know a lot […]and maybe have a relatively decent diet*. *And that all those who actually struggle a lot and fall through*, *on this*, *like*, *they might not be interested or ready to care enough to actually attend such a course* (2D, female, 23, Fine Arts). Another student discouraged a too large focus on environmental sustainability (main topic in this FGD) in the course, believing that this would primarily appeal to those who already know a lot about diet and health. Moreover, it was reflected that the preconception theme (main topic in four FGDs) was somewhat abstract and could therefore be difficult to grasp. It was suggested that most students care more about how diet affect themselves rather than potential future children, and it was mentioned that for most students, the thought of having children would be too far ahead to be thinking about. *If you are a little interested*, *you attend*, *if you do not care*, *you do not care* (2C, female, 23, Health and Sport Sciences). Some students said that their perception of the relevance and utility of *Skills for Life* depended on who was compiling the evidence for the course and what kind of information they delivered. They would not like if the messages conveyed were of a completely different opinion than their own, or if private companies were involved in delivering the course. One student felt that the *Skills for Life* topic were more suitable in primary, secondary or upper secondary school, while others were uncertain whether they would allocate time for it, even though they were interested in attending. Another student remarked that if the course was tailored to enthusiasts and only chosen by a few, she would not go.

#### Lack of appeal

Some students believed that people cannot be bothered to do things unless they get something out of it, e.g. looking good on the CV. *I think very many people cannot be bothered to do things if they do not get anything out of it*, *in a way*, *if it does not look good on the paper or that you have to spend a little extra time at school and could actually go home […]* (6A, female, 23, Health and Sport Sciences). One student thought that unless the *Skills for Life* course provided in-depth knowledge, many students would quit. The course should not feel like *[…] well*, *the same as in secondary school* (11D, male, 19, Engineering and Science). It was mentioned that it might be easy for people to skip the course if there were no compulsory lectures, and one student underlined that it would have to have an aim, e.g., an exam, otherwise she could not bother to go unless she was really concerned about the topic.

#### Too demanding, demotivating, or expensive

Some students were concerned about the possibility that *Skills for Life* might be too demanding or time-consuming when taken in addition to other studies. It was said that setting the level too high in the beginning possibly could induce dropouts, as people might wonder why they should learn the subject so thoroughly, when only for personal use. *I do not like know how many who would bear having an extra*, *a whole extra course in exactly that* (13D, female, 19, Health and Sport Sciences). It was underlined that by delivering more theory than practice, the students might become demotivated, as they also would if the theory was perceived as dry and boring. One student said that she would not bear attending a *Skills for Life* lecture only to sit there, listening, while another student would not skip other lectures to attend *Skills for Life*. Some said that having a poor grade in *Skills for Life* would be very demotivating; signalling that *I definitely do not handle life* (12D, female, 22, Health and Sport Sciences), and some would not attend at all if the course was graded because it might induce stress. One student exclaimed that the studies made her stressed and hindered a healthy diet, and that she regularly skipped making a proper dinner because she had to study. A high fee would be a barrier, and some students would not attend the course without their fellow students or friends.

### Course content and learning outcomes

When questioned more specifically about what they wanted to learn, the students mentioned nutrition knowledge, e.g. regarding a balanced diet, what nutrients and other substances different foods contain, how the food affects the body and how to avoid nutrient deficiency. Furthermore, food preparation skills were highlighted, especially how to cook sustainable, cheap, easy, quick, nutritious, and tasty meals. The students were also interested in learning about sustainable food choices and how to reduce food waste. Some students wanted clarification regarding food myths, the food industry, and the rationale behind the dietary guidelines, and some wanted to learn about planning, shopping lists and making a food budget. Diet in relation to physical activity and exercise was mentioned, as was statements about how to integrate the knowledge and skills in their everyday life; to make healthy lifestyle a habit, and to achieve a balanced diet and a good relationship with food, rather than losing weight and counting calories.

## Discussion

The overall majority of students in this study thought that the idea of a *Skills for Life* course was interesting, relevant and potentially useful for themselves and other students. The participants were surprisingly enthusiastic about the potential of the proposed diet and nutrition course across disciplines and highlighted personal benefits such as increased food preparation skills and improved health and wellbeing. Some also mentioned potential academic benefits such as enhancing their CV, and wider societal benefits such as a healthy population and sustainable food consumption.

Several students claimed that they, and possibly many others would attend the course. This is in line with previous findings showing that college students are interested in learning about healthy eating [32] and the fact that young women especially feel that they need more information and knowledge regarding healthy nutrition and good eating habits [33]. In our sample, 71% of female and 50% of male students highlighted nutrition knowledge as an important learning outcome of *Skills for Life*. Food and health are hot topics in the media and the public debate, where every voice can be heard without any quality proof or peer reviewing. It is not easy to navigate in this jungle of information, especially not for young people who may be following a number of influencers in social media. Many influencers give undocumented advice concerning diet and lifestyle, and private companies that benefit from the health information they provide may buy valuable space for marketing in social media [34]. Malan et al. reported confusing food and nutrition information (e.g. conflicting internet sources and media coverage of dietary guidance) as a challenge for college students to develop and apply food literacy, whereas university courses were described as an opportunity to develop food literacy [35]. Other groups have also described the need for reliable sources of information about healthy nutrition [33]. The wish for “valid” information came up during the FGDs in our study as well, as some students wanted to learn more, and to build capacity to critically assess information online. Critical thinking is one of the previously described life skills [5] that is beneficial in all aspects of life.

In line with the public management developments from the 1980s [36], schools started to put a greater emphasis on high-test scores and advanced academics at the expense of classes in e.g., home economics. The educational theorist E. D. Hirsch’s seminal work on ‘cultural literacy’ and his concern regarding American students without basic life skills has escalated discussions both in Anglo-Saxon and European countries on how to reposition home economics into the curriculum [37–39]. In Europe, OECD (Organisation for Economic Co-operation and Development) has encouraged the mobilisation of knowledge, skills, attitudes and values in the education systems to meet complex demands [40]. Physical and practical skills such as preparing food are highlighted as essential for students’ overall functioning and well-being. In Norway, home economics is the school’s smallest subject when measured in number of classes delivered [41]. Based on their own accounts, several of the participants in our study lacked diet literacy and cooking skills, possibly implying that they represent a generation in a school system where home economics has been diminished in the curriculum over years. This supports the OECD focus on enhancing such knowledge and skills throughout the education system [40], e.g., through home economics and dietary *Skills for Life* courses. Indeed, a previous study detected healthier food choices among college students after completing a personal nutrition course focusing on improving personal nutrition self-efficacy and nutrition behaviour skill-building [42]. Strong et al. suggested that young adults lack planning and self-monitoring skills to maintain healthy behaviours in a college environment [32]. Young people like students comprise a potentially vulnerable group as many of them are moving from living with their parents to taking care of food and cooking by themselves. The idea of offering a dietary *Skills for Life* course is one way of empowering them info “living adult lives”, as one student (2A) described it. Educating and empowering people have been found to be effective ways of promoting health [42–44], but an essential point to achieving this is to take effort in enhancing the likelihood that the students prioritise the course.

It is important to note that the students already had been discussing important food and health issues (i.e., preconception diet in relation to future health, food determinants and sustainable diet) prior to discussing the *Skills for Life* course and hence had achieved a preunderstanding concerning the relevance of such a course. This, combined with the possible group dynamics of the focus groups, where the students may have wanted to appear in a certain way, possibly making it difficult to stand out, makes it understandable that the students seemed mostly positive towards the course. Nevertheless, there were also critical inputs. It is important to investigate the critical voices thoroughly to overcome barriers and increase the probability for students to attend and complete the *Skills for Life* course. The key points were that it is essential that the course provides both personal and academic benefits to get the students to enrol. As the course focuses on diet and cooking, one can argue that–just as many students described it–it will first and foremost benefit their personal life. However, one student (6A, female, 23, Health and Sport Sciences) claimed that the university stood in the way of her healthy diet due to stress and because she skipped dinner because she had to study. If the students through a *Skills for Life* course could learn to plan for and cook easy, cheap, quick and healthy meals, they would probably enhance their diet and health, providing more energy to concentrate while studying, and to endure longer, both regarding personal life, academic performance and future career. Focusing on these benefits in marketing the course might induce interest in originally uninterested students. Furthermore, a broad approach, showing that the course is suitable for all students regardless of prerequisites and study program, delivered by “neutral” actors (i.e. university employees/scientists, not the food industry), and a well-composed course with engaging lectures including both basic and in-depth knowledge combined with practical cooking classes would also have the potential to increase the course’s value and popularity.

### Strengths and methodological limitations

In this study, FGD was chosen as a suitable method of data collection, providing in-depth knowledge of the perceptions and reflections of a sufficiently high number of university students. By conducting FGDs rather than individual interviews, a larger population can be interviewed in the same amount of time, and due to the interaction between interviewees, rich information on both individual and shared opinions on a theme may be obtained [45]. Due to the group dynamics, the type and range of data obtained are often deeper and richer compared to individual interviews [46]. Qualitative research and focus groups are especially suitable when the objective is to explore thoughts, views or attitudes in a group [47], such as in the present article, where the objective was to explore students’ reflections and opinions in relation to a proposed diet and nutrition course.

The fact that the FGDs comprised students of both genders and from all faculties/disciplines at the university increases the representativity of our findings. The gender balance corresponded to that of Norwegian students in general (approximately 60% women and 40% men [48]). Providing a gift card as a motivator for participating in the study rather than relying on interest in diet and nutrition may have contributed to the representativity and thus increased the validity of the findings. The FGDs were conducted at the student’s habitual study environment, which may have helped them relax and express themselves more freely. Moreover, as the moderators were students themselves, the imbalance of power between researchers and students may have been less prominent compared to moderation by more experienced and older researchers. All three postgraduate students were present at all FGDs and they double checked each other’s transcripts afterwards, reducing the probability of errors. Other strengths were the involvement of a team of researchers rather than just one researcher in the process of analysis, and that the article has been written in accordance with COREQ [29] and systematic text condensation [31]. The interpretation of the interview data presented in this paper is only one of many possible interpretations.

We aimed at including 60 students and had to invite as many as 1000 students to get a sufficiently high number of participants. The low response rate (< 6%) may partly be explained by the fact that experience has shown that many students do not check their institutional e-mail regularly. Concerning the validity of our findings, caution must be taken as the students were primed by discussing important food and health issues prior to discussing the *Skills for Life* course. Limitations by conducting FGDs rather than individual interviews are that the students may have refrained from expressing their opinion because they wanted to appear in a socially appropriate way, and that some students might have dominated the discussion. The latter was addressed by the moderators as they tried to guide the FGDs and encouraged quiet students to speak.

## Conclusion and implications

Most students acknowledged the importance of dietary knowledge and skills and the relevance and utility of the proposed *Skills for Life* course, however important barriers such as potential lack of appeal were also described. We believe that young people have the right to be acquainted with strategies and behaviours that promote health for themselves and their prospective children. The students believed that attending a life skills course targeting diet might benefit their personal and academic lives, in addition to yielding societal benefits such as contributing to a sustainable food consumption. Our findings underpin the potential value of offering a tailored diet-related life skills course at university level in line with the role of universities in educating and empowering students to become healthy and literate citizens in a 21^st^ century society. Future development and implementation of life skills courses at university level should be thoroughly evaluated.

## Supporting information

S1 File Interview guide. (PDF)
