# Supplementary material for: “It is really just brilliant to get credits for something that is so important to you!” Skills for Life: University students’ perceptions of a planned dietary life skills course
Source: PLoS One. 2022 Apr 7;17(4):e0260890. doi: 10.1371/journal.pone.0260890 (PMC8989186; doi:10.1371/journal.pone.0260890)
Supplement: S1 File — (PDF) [file pone.0260890.s001.pdf]

Intervjuguide – Skills for Life

Alt vi har snakket om i dette intervjuet i dag kan brukes til å utvikle et emne for studenter som fokuserer på ernæring, helse og matlagingsferdigheter. Emnet skal være valgfritt for alle studenter på universitetet og målet med dette kurset er å styrke studenters kunnskap, holdninger og ferdigheter omkring kosthold og helse. Sett at dette skulle bli et studiepoenggivende emne på UiA som man kunne velge:

- Hva tenker dere om et slikt tilbud?
- Hvordan hadde dere foretrukket at emnet ble lagt opp?
    - Nettbasert, digitalt eller klasserom
    - Praktisk matlaging vs. Teori
        - Fordeling
    - Varighet
        - Varighet på øktene
    - Omfang
        - 5 studiepoeng, kveldskurs, timer i uken
    - Moduler/temaer/innhold
    - Godkjent/ikke godkjent eller karakter?
- Hvilke tema ønsker dere å kunne mer om?
- Hva kan dere for lite om innen mat og ernæring?
- På hvilken måte tenker dere at man burde lære om hvordan kommende foreldres helse og kosthold påvirker deres fremtidige barn?
- Hva skal til for at dere skulle ha deltatt på et slikt emne?

Interview guide – Skills for Life *(translated from Norwegian to English)*

Everything we have talked about in this interview today, can be used to develop a course for students that focuses on nutrition, health, and cooking skills. The course will be optional for all students at the university, and the aim of this course is to strengthen students' knowledge, attitudes and skills regarding diet and health. Suppose that this was to be an elective credit-providing course at UiA:

- What do you think about such an offer?
- How would you prefer the course to be organised?
    - Online, digital or classroom
    - Practical cooking vs. theory
        - Distribution
    - Duration
        - Duration of the sessions
    - Scope
        - 5 credits, evening course, hours a week
    - Modules/topics/content
    - Pass/fail or grades?
- What themes do you wish to know more about?
- What do you know too little about regarding food and nutrition?
- In what way do you think one should learn about how future parents' health and diet affect their future children?
- What would it take for you to attend such a course?
